# Preventing and approaching crises for frail community-dwelling patients through innovative care (PRACTIC): study protocol for a process evaluation of a complex intervention in home care service

**DOI:** 10.1186/s13063-025-08876-w

**Published:** 2025-05-28

**Authors:** Ellen Thea Gjelseth Dalbak, Anette Væringstad, Bjørn Lichtwarck, Janne Myhre, Daniela Holle, Sverre Bergh, Øyvind Kirkevold

**Affiliations:** 1 The Research Centre for Age-Related Functional Decline and Disease, Innlandet Hospital Trust, Ottestad, Norway; 2 Department of Health, Care and Nursing, Faculty of Medicine NTNU, Norwegian University of Science and Technology, Gjøvik, Norway; 3 Faculty of Social and Health Sciences, Inland Norway University of Applied Sciences (INN University), Elverum, Norway; 4 Norwegian Centre for Violence and Traumatic Stress Studies (NKVTS), Oslo, Norway; 5 Department of Nursing, Midwifery and Therapeutic Sciences, Bochum University of Applied Sciences, Bochum, Germany; 6 The Norwegian National Centre for Ageing and Health, Vestfold Hospital Trust, Vestfold, Norway

**Keywords:** Process evaluation, Crisis, Home care service, Mixed methods study, Implementation, Complex intervention, Study protocol

## Abstract

**Background:**

The prevalence of frailty increases with older age. Frail and/or multimorbid patients are at risk for experiencing a crisis. Crises are major stressors for the patient, informal caregivers and formal caregivers and often lead to adverse events and hospitalization. The ongoing effectiveness study in the Preventing and approaching crises for frail community-dwelling patients through innovative care (PRACTIC) study is a cluster randomized controlled trial. This study will test the effectiveness of an adapted version of a biopsychosocial person-centred model, the Targeted Interdisciplinary Model for Evaluation and Treatment of Neuropsychiatric Symptoms (TIME), to prevent and resolve crises for frail community-dwelling people receiving home care services. This current study protocol describes the process evaluation that will be conducted in parallel with the effectiveness study.

The aims of this process evaluation are to explore factors and areas of importance in further implementation of the TIME model in home care services in Norway and to make causal assumptions about the effectiveness or lack of effectiveness of the intervention.

**Methods:**

The process evaluation will use mixed methods and integrate an exploratory and convergent design. To guide the process evaluation, we will use the Practical, Robust Implementation and Sustainability Model (PRISM) and Reach, Effectiveness, Adoption, Implementation, and Maintenance (RE-AIM) frameworks for following and evaluating the intervention with TIME. Data collection from municipal staff and home care service leaders will focus on the RE-AIM dimensions. Qualitative data will be obtained through focus group interviews and local project group meetings and analysed using thematic analyses. Quantitative data will be collected by staff questionnaires and will be analysed using descriptive statistics.

**Discussion:**

The PRACTIC study will enhance innovation in the development of new knowledge and a new approach towards each patient. This process evaluation will allow for a better understanding of the intervention and implementation of the complex TIME intervention in home care services. By analysing the RE-AIM dimensions, we will make causal assumptions about the effectiveness of the intervention. The findings will provide comprehensive knowledge of areas and factors of importance for the implementation of TIME in home care services.

**Trial registration:**

Parent trial NCT05651659 (ClinicalTrials.gov), first registered on 15th December 2022.

**Supplementary Information:**

The online version contains supplementary material available at 10.1186/s13063-025-08876-w.

## Background

The absolute number and proportion of older adults in global populations are witnessing a substantial rise. The number of individuals over 60 years of age is projected to double between 2012 and 2050 [1]. In several Organisation for Economic Co-operation and Development (OECD) countries, the proportion of older people receiving long-term care at home ranges from 50 to 75% [2]. Approximately 110,000 adults aged > 67 years in Norway received home care services in 2022, and among these, 70,000 were ≥ 80 years of age [3]. With an expected doubling of the proportion of adults ≥ 80 years of age in the next 40 years, from 4 to 8%, 215,000 adults aged ≥ 80 years will need home care services in 2060 [4]. Home care services encompass a wide spectrum of responsibilities, ranging from providing practical support in managing patients’ households and daily activities to delivering specialized medical treatments. The home care services in Norway are managed, organised and primarily financed by the municipalities [5]. The municipalities serve a dual role, both in implementing national policy goals and prioritising funding based on local preferences. In other words, they are both state agents and autonomous decision-makers [6]. Home care services can be characterized as a complex organization that relies on the collaboration and interdependence of various sectors to ensure the safety and well-being of individuals residing at home. This collaborative network includes essential contributors such as general practitioners (GPs), specialist health care providers, primary health care workers and the social welfare sector, all playing vital roles in enabling individuals to maintain a secure living environment [7, 8]. Older adults in need of home care services are often frail and multimorbid. Frailty can be defined as “a state of physiological vulnerability with reduced capacity to adapt to and manage external and internal stressors” [9]. The prevalence of frailty is estimated to be 11% among adults aged ≥ 65 years and increases to 50% among those > 80 years of age [10]. Studies have emphasized the relevance and utility of a biopsychosocial definition of frailty, including the terms physical frailty, psychological frailty and social frailty. Combining these three dimensions into a multidimensional concept of frailty promotes the use of targeted multidomain interventions tailored to older adults’ frailty status [11, 12]. Several studies have reported associations between cognitive impairment and frailty [13, 14]. Approximately 100,000 people in Norway have dementia, a number that will double in the next 20–25 years [15–17]. Among persons with dementia receiving domiciliary care, a large proportion have at least one clinically significant neuropsychiatric symptom (NPS), with depression (14%), anxiety (10%) and apathy (10%) being most prevalent [18].

According to crisis theory, crises are defined as “a process where the stressors cause an imbalance requiring an immediate decision that leads to a desired outcome and therefore crisis resolution” [19]. In the Preventing and approaching crises for frail community-dwelling patients through innovative care (PRACTIC) study, crises are defined as “critical challenges and symptoms that demand immediate and new actions”. The triggers and symptoms that give rise to and sustain crises are diverse and differ among patients. These can include depression, inadequate nutritional status, resistance to care, incontinence, neuropsychiatric symptoms and social isolation [19].

Frail and/or multimorbid patients are at risk of crises. The “Mind the Gap report” from the Advisory Board of the Global Forum for Health Care Innovators states that 15–35% are patients with an increasing risk and 1–5% of community-dwelling patients are high-risk patients. Crises often lead to adverse events, the use of coercion and acute institutionalisation [20] and are major stressors for patients, informal caregivers and home care professionals. Furthermore, crises may pose an economic burden to society caused by the increased use of health care services. It is therefore paramount to investigate novel methods for approaching and preventing crises and to improve the quality and efficacy of home care services.

The present study protocol is part of the larger PRACTIC study and describes the design of a process evaluation which is conducted parallel to cluster randomized controlled trial (cRCT) with a participatory action research design. The cRCT tests the effectiveness of an adapted version of a biopsychosocial person-centred model, Targeted Interdisciplinary Model for Evaluation and Treatment of Neuropsychiatric Symptoms (TIME), to prevent and resolve crises for frail community-dwelling people receiving home care services [21]. TIME is a complex intervention that comprises several interactive components [22]. The evaluation of complex interventions is more than testing the effectiveness on a given outcome. The cRCT will therefore be accompanied by a process evaluation, which is the primary purpose of the present study.

### Evaluation of complex interventions

The British Medical Research Council (MRC) guidelines for process evaluation of complex interventions emphasise that process evaluation is an essential part of designing and testing complex interventions [23]. Complex interventions are interventions built up from several components that act interdependently [24]. One of the central issues in the process evaluation of complex interventions is the importance of assessing the fidelity to the intervention, which refers to the degree to which an intervention or programme is delivered as intended [23, 25–28]. This can provide knowledge about why an intervention succeeds or why it fails [26], and furthermore, it can give valuable insight into contextual factors that may influence the outcome [28]. Lastly, a process evaluation can be used to evaluate internal and external validity of the results of an intervention [29]. Internal validity, in this context, refers to the credibility and trustworthiness of the implementation outcomes, ensuring that the observed effects are genuinely due to the intervention and not external factors. External validity highlights the extent to which insights from the process evaluation can be applied in other settings, beyond the immediate study context [29, 30].

### Aims of the process evaluation

The aims of this process evaluation are (a) to explore factors and areas of importance for future implementation of the TIME model in home care services in Norway and (b) to make causal assumptions about the effectiveness or lack of effectiveness of the implementation of the model. These causal assumptions will be presented as a part of a logic model for the intervention. Studies about the process evaluation of complex interventions in home care services for older people are scarce. This study seeks to answer the following research questions: (1) What is the internal validity of the intervention with TIME? (2) What is the external validity of the intervention with TIME? (3) What are the barriers and facilitators for the implementation of the TIME model? (4) What are the costs of implementing and operating the TIME model?

### Implementation of the TIME intervention

To understand the implementation process, it is crucial to gain information about what have been implemented and how the intervention is performed. Thus, a description of the intervention and evaluation of the implementation strategy or procedures for implementation are core elements in the process evaluation [31].

#### TIME intervention

A complex intervention consists of several elements and steps and will involve different parts of an organization and people with different roles. Hawe et al. [32] problematize the evaluation of fidelity of a complex intervention when local adaptations must be performed. The organization of a municipality and its structural factors may influence the implementation of the intervention (contextual factors) [26, 33]. This emphasises the importance of describing the intervention (i.e. the core elements) and the context where the TIME model was implemented and interpreting the fidelity of the intervention. In addition, the implementation strategy should be presented (Table 1).

**Table 1:** Components of the intervention and implementation strategies

Components of the intervention and implementation strategies	Content	Participants (target population)	Duration
Core elements of the intervention			
The assessment phase	PGSI^a^, personal history, ADL^b^ assessment, nutritional assessment Medical history, somatic and psychological assessment and examination	Care staff Patient’s GP	Will vary, but must be done before the reflection meeting
The reflection phase	Based on principles from cognitive behavioural therapy and the ABC method A customised treatment plan is developed. One or more case conferences (CCs) are conducted without supervision	TIME administrators, GPs, managers of the home care services, care staff	One hour A minimum of one CC per patient included within the study period of 6 months
Action and evaluation phase	The treatment plan is implemented and evaluated systematically	Care staff and the patient’s GP	Will vary
Components of the implementation strategy			
Information to the home care services	Information concerning the project and data collection, time frame, inclusion criteria and consent capacity	Managers of home care services, project nurses	Six hours
Information to GPs	Information about the role of the GPs in the project and the TIME model	GPs in the municipality	30–60 min
TIME basic course	Lecture about crises, frailty and complexity Lecture and educational film about TIME Training and roleplay related to TIME	Care staff	One hour One and a half hours 45 min
TIME administrator course	Additional lecture about TIME and assessment tools Training and roleplay related to TIME	TIME administrators	One and a half hour 75 min
Case conferences	Based on principles from cognitive behavioural therapy and the ABC method A customized treatment plan is developed The first CC for the first patient in the municipality is conducted with the support of a specialist registered nurse from the educational team. The rest of the CCs are conducted without external support	TIME administrators, GPs, managers of the home care services, care staff	One hour
Local project meetings	Adapt the implementation process and the TIME model to the local context of the home care services	TIME administrators, GPs, managers of the home care services, staff from the home care services	Two meetings within the study period of 6 moths
Meetings with the educational and training teams	Adapt the education and training of the TIME model to home care services	Research team	Will vary, based on feedback

^a^PRACTIC Goal Setting Interview

^b^Activities of daily living

In a feasibility trial conducted in 2020, the TIME model was found to be useful and feasible for staff in home care services [34]. Modifications have been made after this feasibility pilot trial in the protocol for the effectiveness trial to achieve a more individual goal-oriented primary outcome, broader inclusion criteria and better tailored education and training for home care services [34]. The TIME model is described in detail elsewhere [22]; however, a short description of the core elements that are crucial in the development of a fidelity checklist is given here.

TIME is a multicomponent interdisciplinary model consisting of three overlapping phases. First, the assessment phase where the care staff and the GP collaborate in a comprehensive biopsychosocial assessment. The assessment phase should always include a PRACTIC Goal Setting Interview (PGSI), personal history, medical history, activities of daily living (ADL) assessment, nutritional assessment, medical review and somatic and psychological assessment and examination. The second phase is the reflection phase with interdisciplinary case conferences based on principles from cognitive behavioural therapy and the ABC method, where a customized treatment plan is developed. In the third phase, the treatment plan is implemented and evaluated systematically. The interdisciplinary case conference is essential in the approach towards a crisis.

There are flexible elements in all the phases in TIME. In the assessment phase, there are additional assessment tools that can be used depending on the patients’ symptoms. For example, a dementia assessment can be conducted if there is a suspicion of dementia. If the municipalities are already acquainted with and prefer specific assessment tools, they are free to utilize them. In the reflection phase, the flexible elements are where, when and how often the case conferences are conducted. Who participates in the case conferences is to some extent flexible. It is desirable that those who work around the individual, including the GP, attend if possible. Sometimes it may be appropriate to include family members, and at other times, not. In the evaluation phase, when and where the evaluation takes place is flexible. For instance, the evaluation can be conducted at the GP’s clinic or with a new case conference if necessary.

#### TIME implementation strategy

The implementation strategy aims to add to the pragmatic character of the cRCT. The intervention will therefore mainly be performed by the staff in the municipality and, to a lesser extent, by the research team [35]. Initially, there will be an educational phase where the educational and training team from the Research Centre will be involved. The educational and training team consists of eight specialist health personnel with TIME educational experience.

The staff in the intervention municipality will participate in 4 h of lectures, training and roleplay related to TIME. The lectures will be standardized according to the steps listed in the TIME manual.

The leaders of the home care services in the intervention municipalities will attend these lectures to ensure that these leaders provide support to the staff during the trial. Each staff member in the intervention group will be provided with the TIME manual, which describes the intervention step by step. They will also be given access to an educational film about TIME and a website to support the intervention.

The project nurses will have special responsibility for putting the model into practice based on the manual. These nurses will receive three additional hours of training and be referred to as TIME administrators in the intervention group. After the educational phase, staff in the municipalities will perform the intervention.

The TIME administrators in each municipality have access to support from a specialist registered nurse from the educational and training team to conduct one of the case conferences, preferably their first. The specialist registered nurse will attend the case conference by videoconference and supervise that the core components of TIME comply with and support the conduction of the conference.

Participatory action research in the effectiveness study aims to ensure adaption of the intervention to the local context [36, 37]. Therefore, two local project group meetings will be conducted with each intervention municipality. The local project group, GPs and representatives from the research group will attend these meetings. The main purpose of these meetings is the adaption of the TIME model to the local context to make the model fit with the organisational structure already established in home care services. This adaptation follows a cyclic process: plan, do, check, act and adjust [38].

### Activities connected to the effectiveness study and the process evaluation

All included municipalities will, before randomisation is performed, have a 6-h information meeting about the PRACTIC study. The main purpose of this meeting is to give the home care service information about the project, the data collection, time frame, inclusion criteria and how to assess capacity to consent to participation in the research. Each municipality has chosen two to five project nurses from the home care service, depending on the size of the municipality, which forms the local project group. One of the project nurses, referred to as a research collaborator, will be the main contact point between the local project group and the research team. The GPs will be offered a 30–60-min information meeting about the project. Key staff from the municipality are the project team and the GPs. Their responsibilities in the project in general and in the intervention municipalities as TIME administrators are illustrated in Table 2. All municipalities, independent of the randomisation results, will be reimbursed with 5200 EUR for participating in the project. This reimbursement will to some extent cover time spent on the project and time used for including patients.

**Table 2:** Responsibility of staff from the municipality

Staff from the municipality	Overall responsibility in the PRACTIC study	Intervention municipality
The leader of the home care service	Facilitate the completion of the study	Facilitate implementation Attend local project group meetings
Research collaborator from the home care service	Main link between the research team and the municipality Participate actively in carrying out the study. Main responsibility to ensure that the inclusion of the patients and patient assessments are carried out according to the schedule. Not participating in the clinical work with the included patients, in principle	Facilitate implementation Facilitate the conduct and implementation of the core components of TIME Attend local project group meetings
Project nurses	Participate actively in carrying out the study. Conduct the inclusion of patients according to the inclusion criteria, assess capacity to consent to participation in the research and obtain consent. Conduct the patient assessments according to the schedule	Main responsibility (as TIME administrators) for conducting and implementing the core components of TIME Attend local project group meetings
GPs	Practice as usual regarding the included patients. Physical and mental examination of the included patients if this is deemed clinically relevant	Participate in the core components of TIME Attend local project group meetings

## Methods/design

The trial protocol will follow the guidelines outlined in the Standard Protocol Items: Recommendations for Interventional Trials (SPIRIT 2013) for the conduct and reporting of clinical trials [39].

### Design

In this process evaluation of the TIME model’s intervention and implementation, we will use a mixed methods design where we integrate exploratory and convergent designs according to Fetters et al. [40]. In an exploratory and convergent design, qualitative and quantitative data are collected and analysed during a similar timeframe [41]. The integration approach is that during this timeframe, the data collection and analysis drives changes in the data collection procedures [40]. For example, the protocols for the interviews may be developed and interpreted in light of quantitative findings (e.g. why is it that so few nurses attend the courses?).

To provide a comprehensive description of the complex intervention and implementation strategy, this protocol is inspired by Holle et al.’s [42] study protocol for conducting a process evaluation for a cluster randomised controlled trial.

The process evaluation will be prespecified by the use of the RE-AIM [43–45] and PRISM [31, 46] frameworks to guide the process evaluation. We will use the Reach, Effectiveness, Adoption, Intervention, and Maintenance (RE-AIM) framework [33, 43, 44] for following and evaluating the intervention with TIME. RE-AIM is a widely used system in which MRC guidance is also listed as a possible approach [47] for evaluating complex interventions. RE-AIM also supports the CONSORT items that are relevant for the process evaluation of randomized trials [33, 45]. The Practical, Robust Implementation and Sustainability Model (PRISM) framework, the contextual expansion of RE-AIM, will be used to describe and identify key contextual factors that impact RE-AIM outcomes [31, 46].

The process evaluation will be carried out alongside the effectiveness study between January 2023 and June 2024.

A total of 30 municipalities (clusters) will take part in the cRCT and thus also in the process evaluation. The quantitative part will be performed in all municipalities, but the qualitative interviews will only be carried out in the intervention municipalities. A more detailed description of the inclusion of the home care services from the municipalities is found in the registered cRCT (Trial number: NCT05651659). Given this design, the process evaluation group will know which municipalities are intervention and control municipalities. However, the results of the cRCT will not be known.

## Data and data collection

Collected data will cover the topics answering the RE-AIM dimensions (see Table 4). In addition, we will gather contextual and organisational data to describe and identify key contextual factors that could affect RE-AIM outcomes. The same quantitative data, both at the patient level and staff level, will be collected in the control municipalities. The staff in the control municipalities will also evaluate the progress of patients in their municipalities. Furthermore, we will register costs related to implementation and operational costs. The connection between collected data and the PRISM and RE-AIM frameworks are further developed in the section about “The connection between the RE-AIM elements and the research questions”, below.

### Qualitative data

The qualitative data in this study will be collected by seven focus group interviews with participants and key informants and from local project group meetings. Focus group interviews are suitable for gathering opinions, experiences and attitudes in a collaborative environment, especially to explore experiences and views on health programs and interventions, in this case experiences with the TIME model in home care services [48, 49]. Participants with different roles will be in separate homogeneous groups. Homogenous groups will make it possible to enhance group dynamics and generate constructive associations [48, 49]. There are two planned focus group interviews with staff members who will have participated in the case conferences, two focus group interviews with TIME administrators, two focus group interviews with leaders, and one focus group with GPs. Participants will be recruited through purposive sampling, ensuring sufficient variation in both professional background and the size of the municipalities (small, medium and large). This approach will contribute to achieve adequate information power, ensuring the sample provides relevant and comprehensive data for the study [50]. The topic in these interviews will be how the TIME model was used in the municipalities, barriers and facilitators in the use of the TIME model, and the organizations’ intention to use the TIME model in the future. In addition, qualitative data will be collected from local project group meetings.

To collect process data from the interventions, members of the research team will perform a structured telephone interview with the TIME administrators in each municipality two times with two-month intervals during the 6-month trial. In the interviews it will be used a fidelity checklist that consist of the core elements of TIME (i.e. the assessment phase, the performance of the case conferences, the actions taken and systematic evaluations of these actions) and eventually local adaptions of the intervention.

### Quantitative data

Contextual quantitative data that will be used are listed in Table 3. They will be collected from Statistics Norway (ssb.no). We will also collect organizational data as number of district nurses, number of staff under each district nurse and number in the care staff in the municipality/care district. In addition, we ask seven questions about evaluation of needs for services and how services are allocated, and four questions about the organization of the daily tasks. This will be mapped with a structured telephone interview with leaders of the home care services. Background information about the staff is collected as part of the staff questionnaires.

**Table 3:** Contextual data

**Contextual data from each municipality**
• Organizational model
• Population
• Proportion of population aged 80 or older
• Number of nursing-home beds per inhabitant aged 80 years or older
• Number of persons receiving in home nursing
• Total number of in home nursing provided last year
• Proportion of staff providing in home care with health care education.

The number of staff members in the included municipalities will be registered together with the number who attend the training program. The proportion of staff who attend the TIME training program will be calculated to inform the Reach dimension in the RE-AIM framework. Furthermore, whether there are some differences between the staff who attended the training and those who did not will be evaluated.

Background information about the staff will cover age, sex, education, percentage of full-time employment, number of years of experience in current jobs and employment relationships.

In addition to the background information, three validated questionnaires will be administered to the staff. The first questionnaire, the General Nordic Questionnaire for Psychological and Social Factors at Work regarding mastery and social interactions (QPS-Nordic), will collect information about organizational climate and the care staff perception of leadership behaviours [51, 52]. The second questionnaire, the Approaches to Dementia Questionnaire (ADQ), will assess attitudes towards dementia [53, 54]. The last questionnaire, the Stress of Conscience Questionnaire (SCQ), will measure the degree to which health professionals experience frequently stressful situations in health care [55].

Staff in the municipalities will be requested to answer questionnaires on two occasions, before the intervention (before baseline and randomisation in the RCT) and after 10 months. A reminder will be sent to those who have not responded.

#### The connection between the RE-AIM elements and the research questions

A full description of the data collection and the connection with the RE-AIM elements is provided in Table 4. In this section, we outline the connection between the data that will be collected and our research aims. It is a focus on the RE-AIM elements; however, the contextual data included in the PRISM framework will be used in the interpretation of each of RE-AIM elements.

**Table 4:** RE-AIM elements related to data collection

Data collection	RE-AIM^a^ dimension	Time frame	Respondents
Proportion and characteristics of staff members attending education and training sessions			
Registration forms from the education and training sessions	Reach – staff level	At the start of every session	Attendants of the sessions
Staff ability to prevent and address crises			
Focus group interviews	Effectiveness – staff	After the study period	Staff
ADQ^b^, SCQ^c^		At the start and at the end of the study period	
The adoption of the TIME model by the organization			
Contextual data and telephone interviews with the leaders	Adoption	Before the start of the intervention	Leaders in the municipality
Local project meetings		Twice during the study period	
Fidelity			
Self-developed questionnaire	Implementation	At the end of the study period	Staff
Checklists during the intervention		Two and four months into the study period	
Local project group meetings		Two and four months into the study period	TIME administrators, GPs, managers of the home care services, staff from the home care services
Focus group interviews		At the end of the study period	
Barriers and facilitators			
Seven focus group interviews	All the items in RE-AIM	After the study period (i.e. immediately after all data from patients in the RCT have been collected)	TIME administrators, GPs, managers of the home care services, staff from the home care services
Local project group meetings		Two and four months into the study period	
QPS-Nordic^d^ selected items	All the items in RE-AIM	Before study start	Staff
Cost of the intervention			
Number of hours and individuals attending training, extra hours for case conferences (compared with meetings as usual), extra time spent for data collection (not research data)	Adoption, Implementation	Before and 1–2 months into the study period	Staff

^a^RE-AIM framework: Reach, Effectiveness, Adoption, Implementation, and Maintenance

^b^Approaches to Dementia Questionnaire

^c^Stress of Conscience Questionnaire

^d^General Nordic Questionnaire for Psychological and Social Factors at Work regarding mastery and social interactions (QPS-Nordic)

### External and internal validity of the intervention with TIME

The main measure for internal validity (i.e. a credible connection between the results and the intervention) is the “fidelity” of the intervention (implementation in RE-AIM). As described above, fidelity refers to the degree to which an intervention or program is delivered as intended and we will use data from the process evaluation (from the checklist) and the focus group interviews to analyse fidelity. Since both qualitative data and some quantitative data are collected, we cannot define a cut-off value for fidelity/not fidelity, but we expect to give a good description of the degree of fidelity based on these data. The intervention RCT study aims to have effect on the patients [21]. As present study is a process evaluation of the intervention, and the intervention heavily rely on attitudes and actions of the staff, changes in ADQ scores and/or SCQ scores (“effectiveness” in RE-AIM) will be in the logical path from intervention to effect on the patients. Did the care staff learn anything (self-developed questionnaire about fidelity), did they have a positive attitude towards the intervention (focus group interviews), did the staff attend the training and the case conference, and to what extent was it the staff who attended the case conference who visited the patients?

The evaluation of external validity can to some extend tell how it would be likely to implement in the future in practice outside the context of an RCT, i.e. whether the results can be generalized. External validity depends on several factors. Firstly, the included municipalities, staff and patients must reflect most of the municipalities, staff and patients in Norway, meaning that these factors must be representative of all the target groups. Secondly, it is crucial that the leaders and administrations in the municipalities see the benefit of the intervention; thus, the “adoption” element of RE-AIM will contribute to the evaluation of external validity. Thirdly, the use of resources in both implementing and running the TIME model are relevant data regarding this (need for training, time used for case conferences and the need for external support after implementation). Fourthly, the effect of the intervention and internal validity is of importance. If we cannot show a positive effect of the intervention, it is of no value to implement the intervention elsewhere, at least without modifications based on lessons learned from the process evaluation. In the same way, the dissemination of the model depends on how well the model is implemented in the project.

### Barriers and facilitators of implementation

The focus group interviews and the local project group meetings will provide information on barriers and facilitators for all the RE-AIM dimensions. In addition, QPS-Nordic data about organizational climate and leadership will enlighten factors that inhibit or facilitate the implementation process.

### Costs related to the intervention and implementation

The leaders of the home care services will be asked to complete a form detailing the expenses associated with the TIME education (including both the TIME basic course and TIME administrator course). This will provide information on costs for hiring substitute staff and any additional payments made to employees who were on leave but attended the training. Additionally, costs related to the educational and training team, such as salaries for preparation time and the time spent delivering the training, will also be documented. These represent the “one-time” costs associated with the implementation. Regarding the operating costs, we will use checklists to examine case conferences, including the number of meetings held, as well as how wany and which professionals participated in these meetings. We will also investigate whether these meetings are held in addition to regular meetings within the service or as part of the usual workflow.

The evaluation of costs related to the intervention and implementation, in addition to any operational costs, will provide important information on adoption and implementation in RE-AIM. None of the elements in RE-AIM explicitly include cost; nonetheless, cost can influence adoption, implementation and maintenance by providing insights into the resource requirements at each stage. Costs are also of importance in regard to the possibility of disseminating a new intervention or care model beyond the frame of a trial. In this way, cost informs the pragmatic character of the trial and its external validity.

### Analysis

#### Mixed method analysis

Integration of data sources and analyses is the key to mixed method analyses [56], not only in the discussion but also in the data collection, analysis and display of the results as part of the presentations [57, 58]. To do this, we will apply a “process-oriented logic model for integrated analysis” and follow the strategies described and illustrated by Bazeley [56]. Based on the “Logic Model Development Guide” published by W.K. Kellogg Foundation [59], we have developed a logic model in PRACTIC [see addition file 1]. Even though we will apply an integrated method for analysing the data, widely recognized methods must be used in the detailed analysis of both the quantitative and qualitative data before the decision of how to integrate analyses can be made [56]. Thus, both inductive approaches and deductive approaches will be used to enlighten the elements in both the PRISM and RE-AIM frameworks. This will apply to all the research questions.

#### Qualitative data

For the qualitative data, thematic content analysis will be used. Thematic content analysis is a method for identifying, analysing and reporting patterns and themes in qualitative data [60]. The aim is to provide a systematic description of both the manifest and latent content of the data and ultimately to evolve new concepts and understanding of phenomena. First, interviews will be transcribed. Researchers will generate codes, and these codes will be systematically restructured into meaningful patterns that will be reorganized into meaning-based patterns to accentuate the meaning content of the data.

#### Quantitative analysis

All data will be analysed using descriptive statistics [61]. The data will be presented as frequencies and percentages for categorical variables and means (standard deviations) for continuous variables. The normality of continuous variables will be assessed graphically. If necessary, skewed data will be transformed. To explore possible determinants (e.g. age, sex, education, work experience, work position and score on QPS-Nordic subscales) of effects at the staff level (stress (SCQ) and attitudes towards dementia (ADQ)), we will use regression models adjusting for possible cluster effects at the municipal level.

#### Missing data

For the scale variables (QPS-Nordic, SCQ and ADQ), missing values will be imputed by random numbers drawn from the distribution generated for each item. The imputation will be performed only for cases with at least 50% of items on the scale available. Other categorical items, such as sex and level of education, will not be imputed.

#### Analysis of costs

Cost data will be collected and analysed regarding costs that can be attributed to the implementation of the TIME model (potential “one-time” costs) and costs that will arise during the application of the model (operating costs).

## Discussion

The process evaluation of the PRACTIC study complements the effectiveness study by providing insights into the intervention and implementation process of the TIME model in home care services in Norway. Both the PRISM and RE-AIM frameworks are favourable frameworks for the evaluation of this complex intervention and implementation with TIME [46, 47]. The use of these frameworks will provide an understanding of outcomes that influence the ability of the TIME model to prevent and resolve crises for frail community-dwelling people receiving home care services and the contextual factors that influence these outcomes [62]. Assessing internal validity is of great importance as it establishes a reliable link between the outcomes and the intervention, thereby yielding insights into the fidelity of the implemented approach. An evaluation of a pragmatic trial of effectiveness must be aware of the potential weakness and consequences of implementation errors [63]. Gathering comprehensive information about the implementation process and factors that may influence this process is a strength of this study.

One limitation of this process evaluation is the time constraint; we will not obtain information regarding the maintenance of the TIME model in home care services once the 6-month intervention is concluded. Maintenance, both at the individual and organization-community levels, is a major challenge [44], and it would have been intriguing to determine what happens after the study period is over.

The process evaluation of the cRCT does not involve any patients or next of kin; thus, we have not involved user representatives in this part of the PRACTIC study. However, the cRCT involves patients, and a user group is established for the cRCT, this is described elsewhere [21]. Staff in the municipal healthcare in the involved municipalities participates in the development and the adaption of the intervention through the participatory action research; however, they are not involved in the planning of the process evaluation. This may be a weakness.

## Trial status

The trial was initiated in 2022 and will be completed by mid-2024. The recruitment of municipalities was completed at the end of the summer of 2022. The first patient was recruited on the 9 th of January 2023, and the last follow-up visit for the last included patient will be in April 2024. Data collection from the participating staff will be completed by the end of 2024. The results will be reported in 2025 and 2026 according to protocol version 2 (ETGD, ØK, 20 th of December 2022).

## Supplementary Information

Additional file 1. MS-WORD-format “Additional file 1.docx”. Logic model. Table describing the logic model.

Additional file 2. MS-WORD-format “Additional file 2.docx”. Participant consent form. The participant consent for – translated from Norwegian to English.

## Data Availability

A list of the variables in the datasets is available on the PRACTIC website, www.practic.no. The data collected in the study is not available to the public.

## Abbreviations

ADQ: Approaches to Dementia Questionnaire
MRC: British Medical Research Council
PGSI: PRACTIC Goal Setting Interview
PRACTIC: Preventing and approaching crises for frail community-dwelling patients through innovative care
PRISM: Practical, Robust Implementation and Sustainability Model framework
QPS-Nordic: General Nordic Questionnaire for Psychological and Social Factors at Work regarding mastery and social interactions
RE-AIM: Reach, Effectiveness, Adoption, Intervention, and Maintenance framework
SCQ: Stress of Conscience Questionnaire
TIME: Targeted Interdisciplinary Model for Evaluation and Treatment of Neuropsychiatric Symptoms
