# Supplementary material for: Preventing and approaching crises for frail community-dwelling patients through innovative care (PRACTIC): study protocol for a process evaluation of a complex intervention in home care service
Source: Trials. 2025 May 28;26:178. doi: 10.1186/s13063-025-08876-w (PMC12121081; doi:10.1186/s13063-025-08876-w)
Supplement: Supplementary file 1 — Additional file 1. MS-WORD-format “Additional file 1.docx”. Logic model. Table describing the logic model. [file 13063_2025_8876_MOESM1_ESM.docx]

| Logic Model Template |  |
| --- | --- |

| **Resources / input** | **Activities** | **Outputs** | **Outcomes** | **Impact** |
| --- | --- | --- | --- | --- |
| *Resources are needed to support the solutions the program proposes; activities depend on resources.* | *Activities are those actions planned to bring about intended results.* | *Outputs provide evidence of service delivery. They describe the size and scope of what the program delivers or produces.* | *Outcomes in a logic model are the short-term and long-term results.* | *Impact is the set of changes you expect as a result of your activities, 7-10 years after the work is underway.* |
| Funding from the home care service to support the education program (this entails three days of identical training educational sessions scheduled after lunch, ensuring all staff can participate).  Funding for the educational and training team to provide the educational program (three days plus one follow-up meeting).  Website to support the project and the home care services in the use of TIME.  One 6-hour information meeting about the project with managers of home care service and project nurses. Information concerning the project and data collection, time frame, inclusion criteria and consent capacity. | **Educational program**  4-hour educational program for the entire care staff  Content: Lecture about crises, frailty and complexity. Lecture and educational film about TIME. Training and roleplay related to TIME.  3-hour extra educational program for the TIME administrators (implementation champions)  Content: Additional lecture about TIME and assessment tools. Training and roleplay related to TIME.  The first case conference in the municipality is conducted with the support of a specialist registered nurse from the educational team.  On a regular basis the TIME administrators (implementation champions)  and leader of home care service is responsible for continuing and implementing TIME.  **Local project meetings**  Adapt the implementation process and the TIME model to the local context of the home care services. Two meetings within the study period of six months.  **Meetings with the educational and training teams**  Adapt the education and training of the TIME model to home care services. Number of meetings will vary, based on feedback. | **Interdisciplinary assessment and follow-up service of functional impairment in the user group** Structured assessment through a holistic approach based on multimorbidity and functional impairments. Systematic cooperation and common approach with the GPs and other relevant health care workers in the municipality (e.g. physiotherapist, occupational therapist).  Increased individual goal achievement to resolve or reduce the challenges of the crises.    **Case conferences**  Established on a regular basis as a part of the approach and prevention to crises for frail community-dwelling patients.  Common responsibility of the leader, GP and care staff.  Integrate the patient’s perspective through use of PGSI or with the next of kin participation. | **Changes towards a comprehensive and holistic approach to crises for frail community-dwelling patients**  Structure reduces instability.  Participation in decision making for both the care staff, patient, next of kin, the GP, leaders and other relevant health care workers in the municipality.  A shared visual presentation display enhances mutual understanding and commitment.  **Patients’ outcomes**  Better quality of life.  Reduction of neuropsychiatric symptoms and pain.  Enhanced daily living activities.  Reduction of distress perceived by the next of kin.  **Care staff’s outcomes**  Increased use and knowledge of relevant assessment tools.  Increased knowledge about crises, frailty and complexity.  Better trust in their skills and knowledge.  Improving the approaches to crisis may reduce the use of specialist healthcare services. | A cultural change from a mainly task-oriented service based on failure in activities of daily life, to an interdisciplinary assessment and follow-up service of functional impairment in the user group. |

Reference: W. K. Kellogg Foundation. "Logic model development guide." *Michigan: WK Kellogg Foundation* (2004). Available with other resources at:

<https://wkkf.issuelab.org/resource/logic-model-development-guide.html>
