# Supplementary material for: Preventing and approaching crises for frail community-dwelling patients through innovative care (PRACTIC): study protocol for a process evaluation of a complex intervention in home care service
Source: Trials. 2025 May 28;26:178. doi: 10.1186/s13063-025-08876-w (PMC12121081; doi:10.1186/s13063-025-08876-w)
Supplement: Supplementary file 2 — Additional file 2. MS-WORD-format “Additional file 2.docx”. Participant consent form. The participant consent for – translated from Norwegian to English. [file 13063_2025_8876_MOESM2_ESM.docx]

Consent form for participation in the **PRACTIC** study

This is an invitation for you to participate in a research study on the prevention and approach to crises in vulnerable home-dwelling patients. The study involves the development and testing of an interdisciplinary assessment and reflection model (TIME model). The TIME model is an assessment and reflection model that provides a comprehensive approach to assessing behavioural and psychological symptoms in individuals with dementia. In this study, we aim to examine the use of a customized version of the TIME model as a tool for home care staff.

A total of 30 municipalities are participating in the research study, with half of them using the TIME model after training (intervention municipalities), and the other half continuing their usual practices (control municipalities). After the study is completed, control municipalities will have the opportunity to receive training in the TIME model. The Center for Age-Related Functional Impairment and Disease (AFS), Sykehuset Innlandet, is responsible for the study and all information collected. The study results will be published as scientific articles. All information about you will be anonymized, meaning no participants will be recognizable in these publications.

WHAT DOES participation in THE STUDY mean FOR YOU?

As an employee in an intervention municipality, we would like to gather your experience with participation in the study. You are invited to participate in a focus group interview, along with employees from 6 – 10 other intervention municipalities. The main themes for the focus group interview are participation and practical implementation, resources, and services after participation in the study. There will also be an opportunity to address other relevant topics.

The focus group interview will take approximately 2 hours. Audio recordings and notes will be taken during the session.

Potential benefits and drawbacks

Participation in the focus group interview will contribute to increased knowledge about the process of implementing the TIME model in home care. The experiences will be used to assess whether the results from PRACTIC can be transferred and utilized in other municipalities.

Participation will involve additional work in terms of setting aside time for the interview.

What happens to your information?

The information recorded about you will only be used as described in the project's purpose, without directly identifiable details. A code will link you to your information through a name list. Only approved personnel associated with the project will have access to the name list and the ability to trace it back to you. It will not be possible to identify you in the results of the project when they are published. You have the right to access the information recorded about you and the right to have any errors in the information corrected. If you wish to access this information, you are entitled to receive it within 30 days. You can complain about the processing of your information to the Norwegian Data Protection Authority (Datatilsynet) and the institution's data protection officer. The information will be stored on a secure research server at Sykehuset Innlandet. The information will be presented in a scientific research article. It will not be possible to identify you in the results of the study when they are published. The project leader is responsible for the daily operation of the research project and ensuring the secure treatment of your information. Information about you will be anonymized. Information about you will be kept for five years after the project's conclusion for control purposes.

Voluntary participation and withdrawal of consent

Participation in the study is voluntary. If you choose to participate, you will sign the consent form on the last page. You can withdraw your consent at any time and for any reason without any negative consequences. If you withdraw from the study, you can request the deletion of collected information unless the information has already been included in analyses or used in scientific publications.

If you later wish to withdraw or have questions about the study, you can contact the project leader (see contact information below).

Approval

The study is registered and approved by the Data Protection Officer at Sykehuset Innlandet, case number: 27582200. Project leader Sverre Bergh is responsible for data protection in the study. We process information based on your consent.

Contact information

If you have questions about the study or wish to withdraw your participation, you can contact Øyvind Kirkevold, email address oyvind.kirkevold@aldringoghelse.no, or PhD candidate Ellen Thea Gjelseth Dalbak, email address ellen.thea.gjelseth.dalbak@sykehuset-innlandet.no.

If you have questions about data protection in the study, you can contact the Data Protection Officer at Sykehuset Innlandet: Personvernombudet@sykehuset-innlandet.no. The Norwegian Data Protection Authority's email address is [postkasse@datatilsynet.no](mailto:postkasse@datatilsynet.no).

- Consent for participation in the practic study

i want to participate in the study

| Place and date | Participants name |
| --- | --- |
|  |  |
|  | Participants name in printed letters |

- Consent form for participation in the **PRACTIC** study

This is a request for your participation in a research study on the prevention and management of crises in vulnerable home-dwelling patients. The study involves the development and testing of an interdisciplinary assessment and reflection model called the TIME model, aimed at preventing and addressing crises in vulnerable home-dwelling patients. The TIME model is initially an assessment and reflection model that provides a comprehensive approach to assessing behavioural and psychological symptoms in individuals with dementia. In this study, we aim to investigate the use of an adapted version of the TIME model as a tool for home care staff.

A total of 30 municipalities are participating in the research study, with half of them using the TIME model after training (intervention municipalities), and the other half continuing with their usual practices (control municipalities). After the study is completed, control municipalities will have the option to receive training in the TIME model. All permanent employees and substitutes with over 50% employment at the start of the study are invited to participate. The Center for Age-Related Functional Impairment and Disease (AFS), Sykehuset Innlandet Hospital Trust, is responsible for the study and all information collected. The study results will be published as scientific articles. All information about you will be anonymized, and participants will not be identifiable in these publications.

WHAT DOES participation in THE STUDY mean FOR YOU?

In the study, some municipalities will receive training in the TIME model, while others will not receive such training and will serve as control municipalities. Neither you or the municipality will know whether the municipality will be a control municipality or a municipality receiving TIME model training until the study begins.

If you choose to participate in the study, it means you will fill out a questionnaire at 10-month intervals. It will take approximately 15-25 minutes to complete the questionnaire. The questionnaire includes questions about your education, work experience, job position, attitudes towards dementia, moral stress, and psychological and social factors in your work.

Potential benefits and drawbacks

Participation in the study will involve education and guidance, potentially leading to increased competence for yourself and your colleagues. Insights from the study may help develop a tailored version of the TIME model as a tool for home care, ultimately contributing to improved quality of care and treatment for patients receiving home nursing.

Participation will entail additional work in the form of questionnaire completion.

What happens to your information?

The information recorded about you will only be used as described in the project's purpose, without directly identifying information. A code links you to your information through a name list. Only authorized personnel involved in the AFS project will have access to the name list and can connect it back to you. It will not be possible to identify you in the results of the project when they are published. You have the right to access the information recorded about you and the right to have any errors in the information corrected. If you wish to access this information, you are entitled to receive it within 30 days. You can also file a complaint about the handling of your information with the Norwegian Data Protection Authority (Datatilsynet) and the institution's data protection officer. The information will be stored on a secure research server at Sykehuset Innlandet Hospital Trust. The information will be presented in scientific research articles. It will not be possible to identify you in the results of the study when they are published. The project leader is responsible for the daily operation of the research project and the secure handling of your information. Information about you will be kept for five years after the project's conclusion for control purposes.

Voluntary participation and withdrawal of consent

Participation in the study is voluntary. By completing the attached questionnaire, you consent to participate in the study. The questionnaire should be returned in the enclosed prepaid envelope within 14 days. You can withdraw your consent at any time and for any reason without any negative consequences. If you withdraw from the study, you can request the deletion of collected information unless the information has already been included in analyses or used in scientific publications.

If you later wish to withdraw or have questions about the study, you can contact the project leader (contact information below).

Approval

The study is registered and approved by the Data Protection Officer at Sykehuset Innlandet, case number: 23928741. Project leader Sverre Bergh is responsible for data protection in the study. We process information based on your consent.

Contact information

If you have questions about the study or wish to withdraw your participation, you can contact Professor Øyvind Kirkevold, email address oyvind.kirkevold@aldringoghelse.no, or PhD candidate Ellen Thea Gjelseth Dalbak, email address ellen.thea.gjelseth.dalbak@sykehuset-innlandet.no.

If you have questions about data protection in the study, you can contact the data protection officer at Sykehuset Innlandet: Personvernombudet@sykehuset-innlandet.no. The Norwegian Data Protection Authority's email address is postkasse@datatilsynet.no.
